# Supplementary material for: Organic–Inorganic Hybrid Device with a Novel Deep-Blue Emitter of a Donor–Acceptor Type, with ZnO Nanoparticles for Solution-Processed OLEDs
Source: Nanomaterials (Basel). 2022 Oct 28;12(21):3806. doi: 10.3390/nano12213806 (PMC9654309; doi:10.3390/nano12213806)
Supplement: Supplementary file 1 [file nanomaterials-12-03806-s001.zip › nanomaterials-1948726-supplementary.pdf]

Supporting information

Organic–Inorganic Hybrid Device with a Novel Deep-Blue Emitter of a Donor–Acceptor Type, with ZnO Nanoparticles for Solution-Processed OLEDs

Seokwoo Kang [1,†], Raveendra Jillella [1,†], Sunwoo Park [1,†], Sangshin Park [1], Joo Hwan Kim [1], Dakyeung Oh [2], Joonghan Kim [2] and Jongwook Park [1,*]

[1] Integrated Engineering, Department of Chemical Engineering, Kyung Hee University, Yongin 17104, Korea
[2] Department of Chemistry, The Catholic University of Korea, Bucheon 14662, Korea
* Correspondence: jongpark@khu.ac.kr
† Seokwoo Kang, Raveendra Jillella, and Sunwoo Park equally contributed to this work as first authors.

General information

Reagents and solvents were purchased as reagent grade and used without further purification. Analytical TLC was carried out using a Merck 60 F254 silica gel plate, and column chromatography was performed using Merck 60 silica gel (230-400 mesh). ^{1}H-NMR spectra were recorded on Bruker Avance 300 spectrometers.

The fast atom bombardment (FAB+) and electron ionization (EI+) mass spectra were recorded on JMS-600W, JMS-700, 6890 Series and Flash 1112, Flash 2000 Optical UV-Vis absorption spectra were obtained using a Lambda 1050 UV/Vis/NIR spectrometer (Perkin Elmer). A Perkin-Elmer LS55 (Xenon flash tube) luminescence spectrometer was used to perform PL spectroscopy. Absolute photoluminescence quantum yield (PLQY) values were obtained by using Quantaurus-QY (Hamamatsu). The transient PL was measured with a Quantaurus-Tau fluorescence life time measurement system. The glass transition temperatures (T_g) of the compounds were determined by differential scanning calorimetry (DSC) under a nitrogen atmosphere using a DSC 4000 (Perkin Elmer). T_d values of the compounds were measured with thermogravimetry analysis (TGA) using a TGA4000 (Perkin Elmer). Atomic Force microscopy (AFM) images were obtained using a D3100 microscope (Veeco). Photo images were taken using confocal microscopy (OLYMPUS, IX83, FV3000). HOMO energy levels were determined using ultraviolet photoelectron yield spectroscopy (Riken Keiki AC-2). LUMO energy levels were derived from the HOMO energy levels and the band gaps. SEM images of the ZnO layer were obtained using a JSM-7600F instrument (JEOL). TEM images of the ZnO nanoparticles were obtained using a Tecnai G2 F30 S-Twin (FEI, 300 kV). X-ray diffraction (XRD) patterns of the ZnO layer were recorded using a SmartLab 9 kW diffractometer (Rigaku, target material: Cu, λ = 0.15406 nm). Particle size distribution of the ZnO nanoparticles were obtained using a ELS-2000ZS (Otsuka).

Fabrication procedure of solution-processed OLEDs

ITO glasses (25 mm × 25 mm) were ultrasonically washed in, sequentially, acetone, ethanol, deionized water, and isopropyl alcohol and then dried in an oven at 80 °C. The ITO substrates were further cleaned with UV–ozone cleaner for 10 min before the spin-coating process. In forward devices, a 40 nm-thick hole injection layer (HIL) was prepared by spin-coating using poly(3,4-ethylenedioxythiophene) polystyrene sulfonate (PEDOT:PSS, Clevios PVP AI4083) at 4000 rpm for 60 s and baking at 140 °C for 20 min. A 15 nm-thick hole transport layer (HTL) was prepared by spin coating using poly(9-vinylcarbazole) (PVK, 6 mg/1 mL of chlorobenzene) at 3000 rpm for 40 s and baking at 150 °C for 30 min. After cooling to room temperature, the emitting materials (30 wt% in toluene) were spin-coated at 2000 rpm for 60 s. After annealing at 70 °C for 5 min, the prepared substrates were transferred to a thermal evaporation chamber. The chamber was evacuated to 2.0×10^{-5} torr, and a 40 nm-thick layer of 1,3,5-tris(1-phenyl-1*H*-benzimidazol-2-yl)benzene (TPBi, Lumtec, Taiwan), 1 nm-thick layer of LiF, and 200 nm-thick layer of Al were sequentially evaporated. In inverted devices, ZnO (Avantama N-10, nano particle size of 23.5 nm, polydispersity index of 0.15) as an electron injection layer (EIL) was deposited by spin coating at a speed of 3500 rpm for 60 s and baking at 150 °C for 30 min, whereas 0.25 wt% PEI (Sigma-Aldrich) in H_2O as the ETL and IL was spin-coated at a speed of 5000 rpm for 60 s followed by an annealing for 10 min at 130 °C. The emitting layer (EML) process was the same as for the forward devices. After cooling to room temperature, the prepared substrates were transferred to a thermal evaporation chamber. The chamber was evacuated to 2.0×10^{-5} torr, and a 20 nm-thick layer of TAPC (Lumtec, Taiwan), 10 nm thick layer of MoO_3, and 200 nm-thick layer of Al were sequentially evaporated. The *I*–*V*–*L* characteristics and EL spectra and CIE color coordinates of the solution process OLEDs were measured using a Keithley 2400 SMU sourcemeter and CS-1000A spectroradiometer (Konica-Minolta) at room temperature under ambient conditions. These instruments were operated using the program provided by McScience.

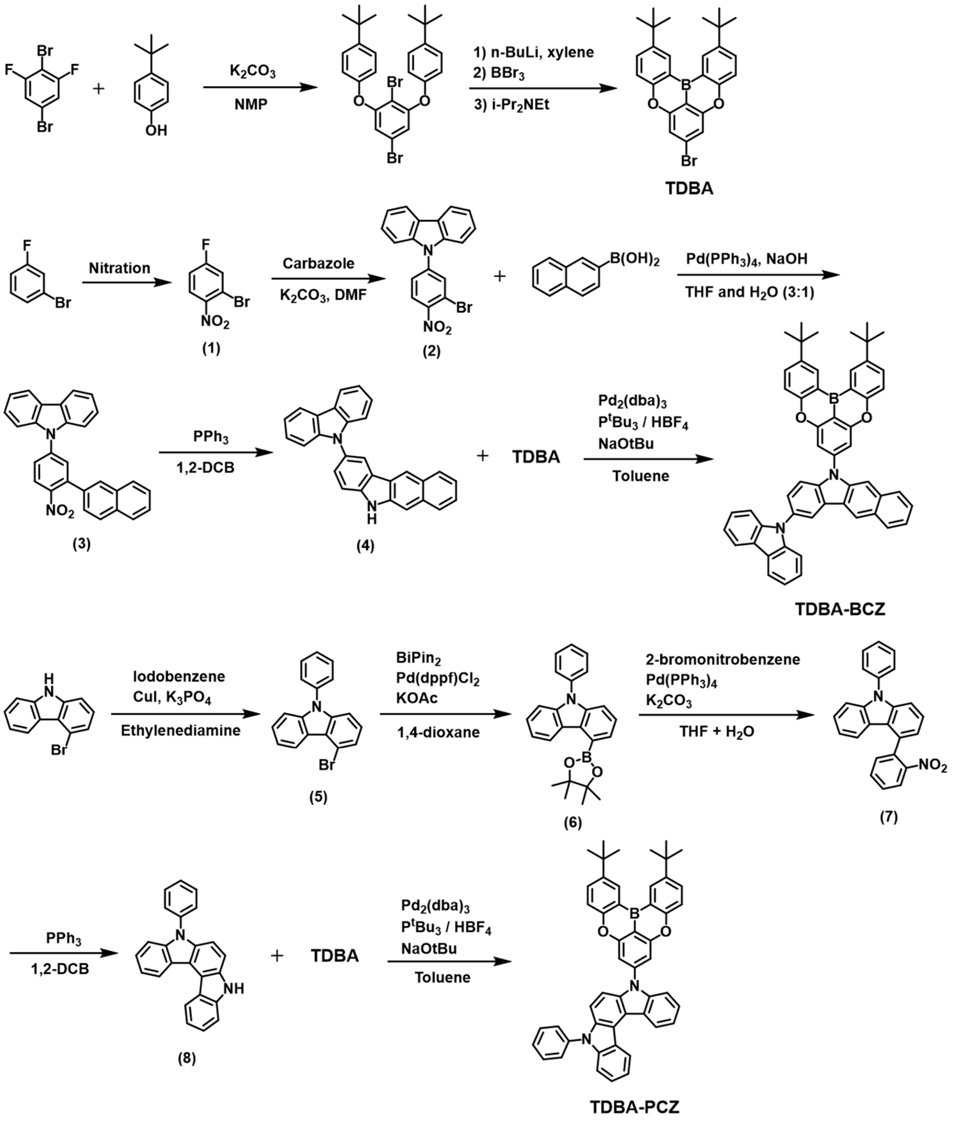

Figure. S1 Synthetic routes for the newly synthesized materials.

Theoretical calculations

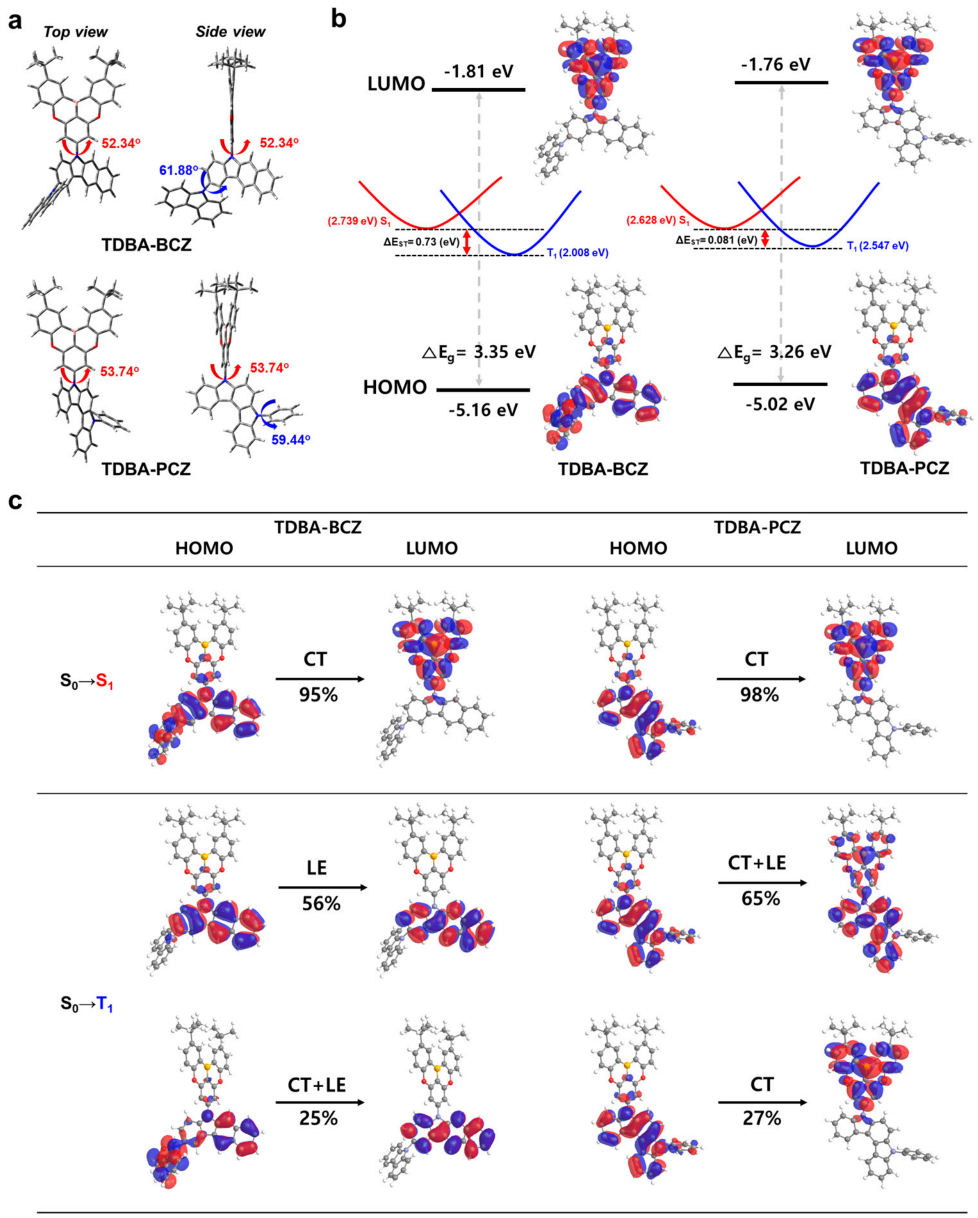

Figure. S2 (a) Optimized structures, dihedral angles, (b) HOMO/LUMO energy levels, and (c) contributions in the electronic transition states of TDBA-BCZ and TDBA-PCZ.

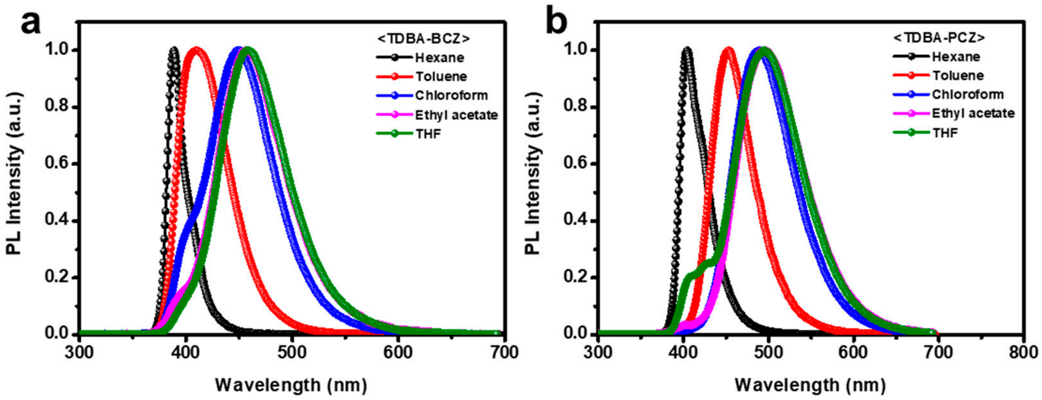

Figure. S3 PL spectra recorded under different solvent conditions for (a) TDBA-BCZ and (b) TDBA-PCZ.

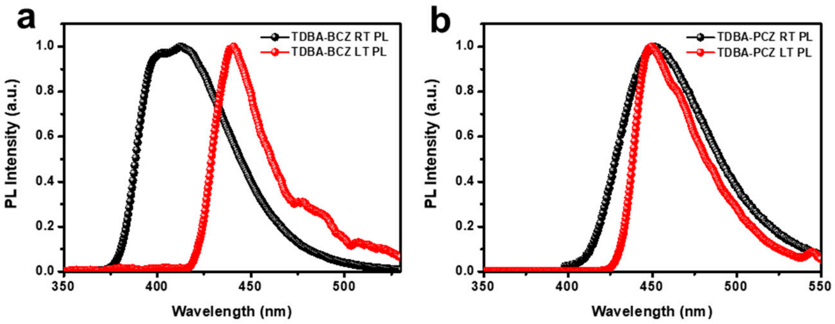

Figure. S4 PL spectra acquired under room temperature (RT) and low-temperature (LT) conditions for TDBA-BCZ (a) and TDBA-PCZ (b).

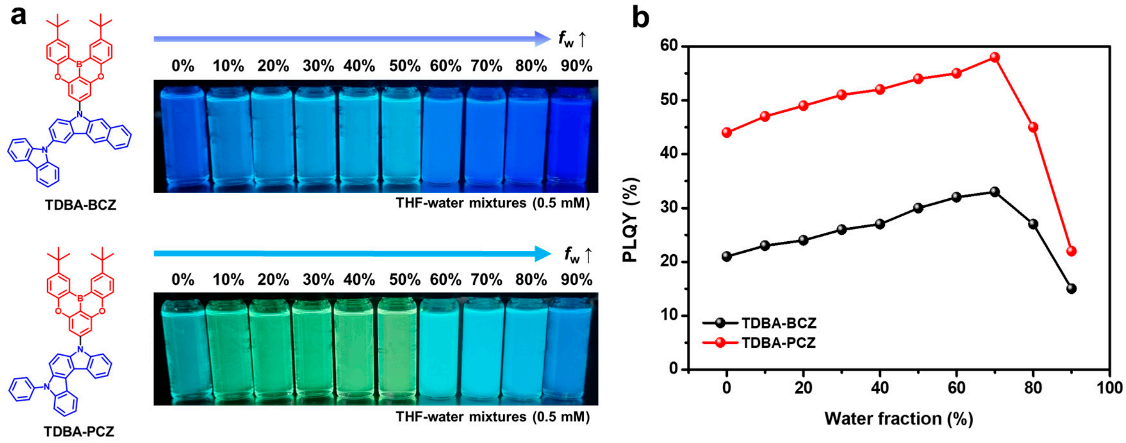

Figure. S5 (a) Photographs and (b) PLQY change graph of THF–water mixtures according to different water fractions (f_w) (0, 10, 20, 30, 40, 50, 60, 70, 80, and 90%) under 365 nm UV irradiation for TDBA-BCZ and TDBA-PCZ.

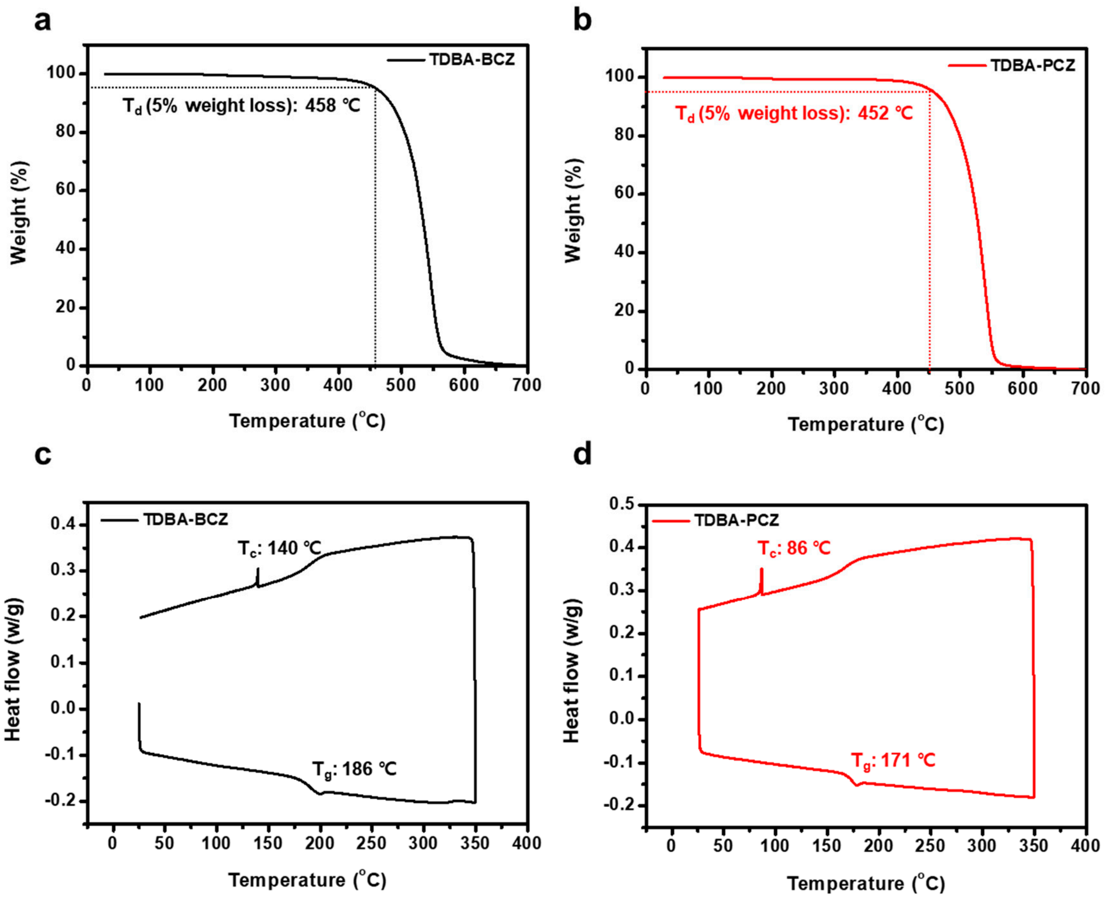

Figure. S6 (a), (b) TGA and (c), (d) DSC curves of TDBA-BCZ and TDBA-PCZ.

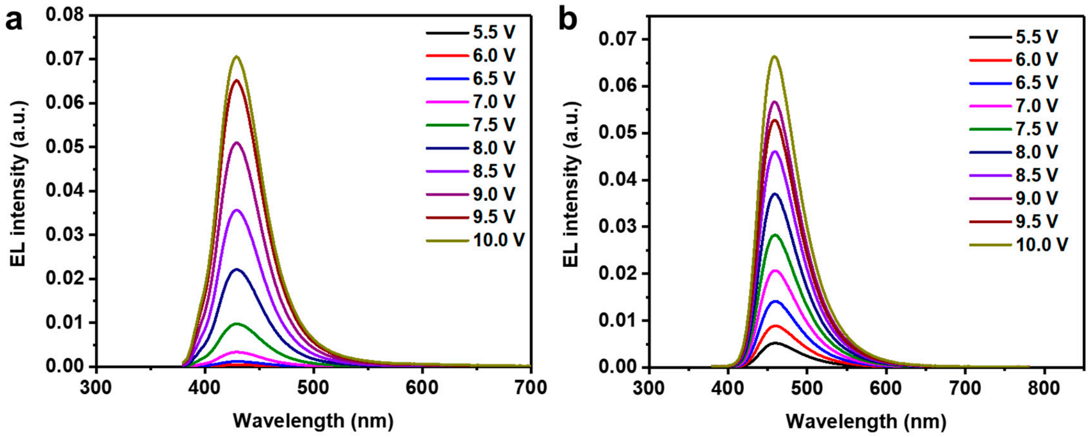

Figure. S7 EL spectra of (a) mCP:30 wt% TDBA-BCZ, (b) mCP:30 wt% TDBA-PCZ at different applied voltages.

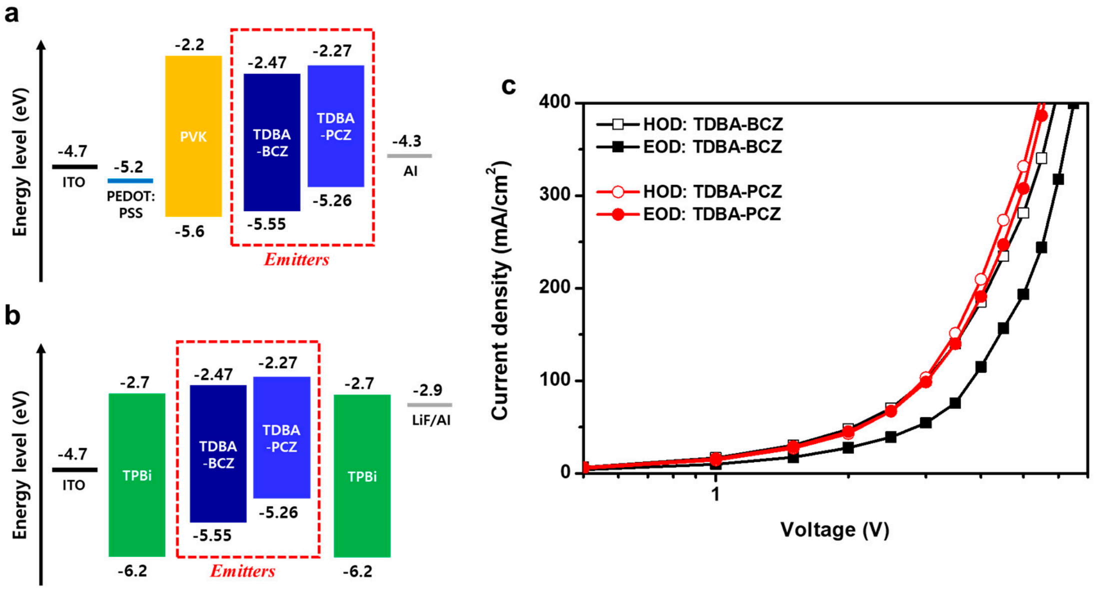

Figure. S8 (a) Hole-only device (HOD) and (b) electron-only device (EOD) structures, and (c) the current density–voltage (*J*–*V*) characteristics for TDBA-BCZ and TDBA-PCZ.

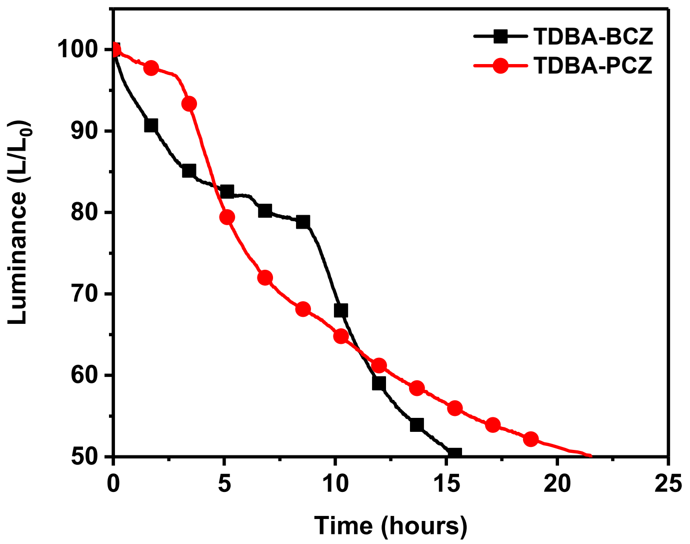

Figure. S9 Operational stability of TDBA-BCZ and TDBA-PCZ

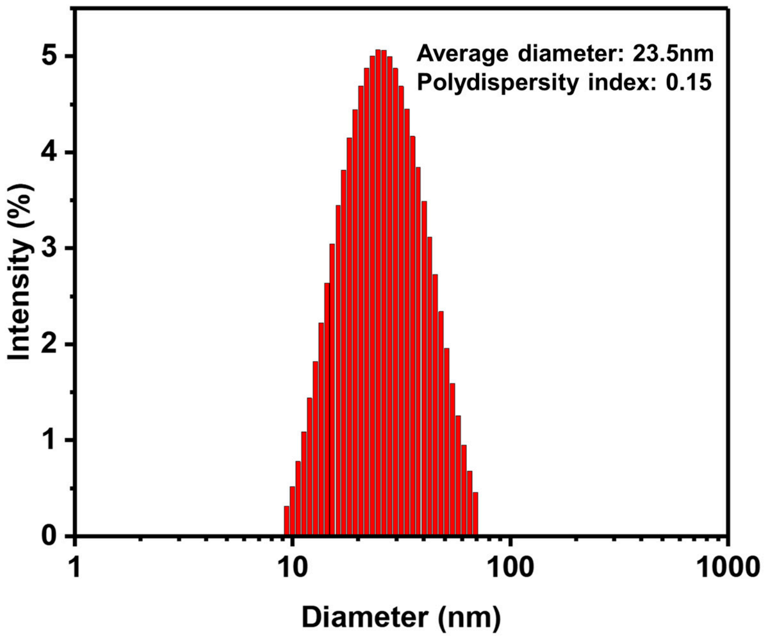

Figure. S10. Particle size distribution curve of ZnO nanoparticles

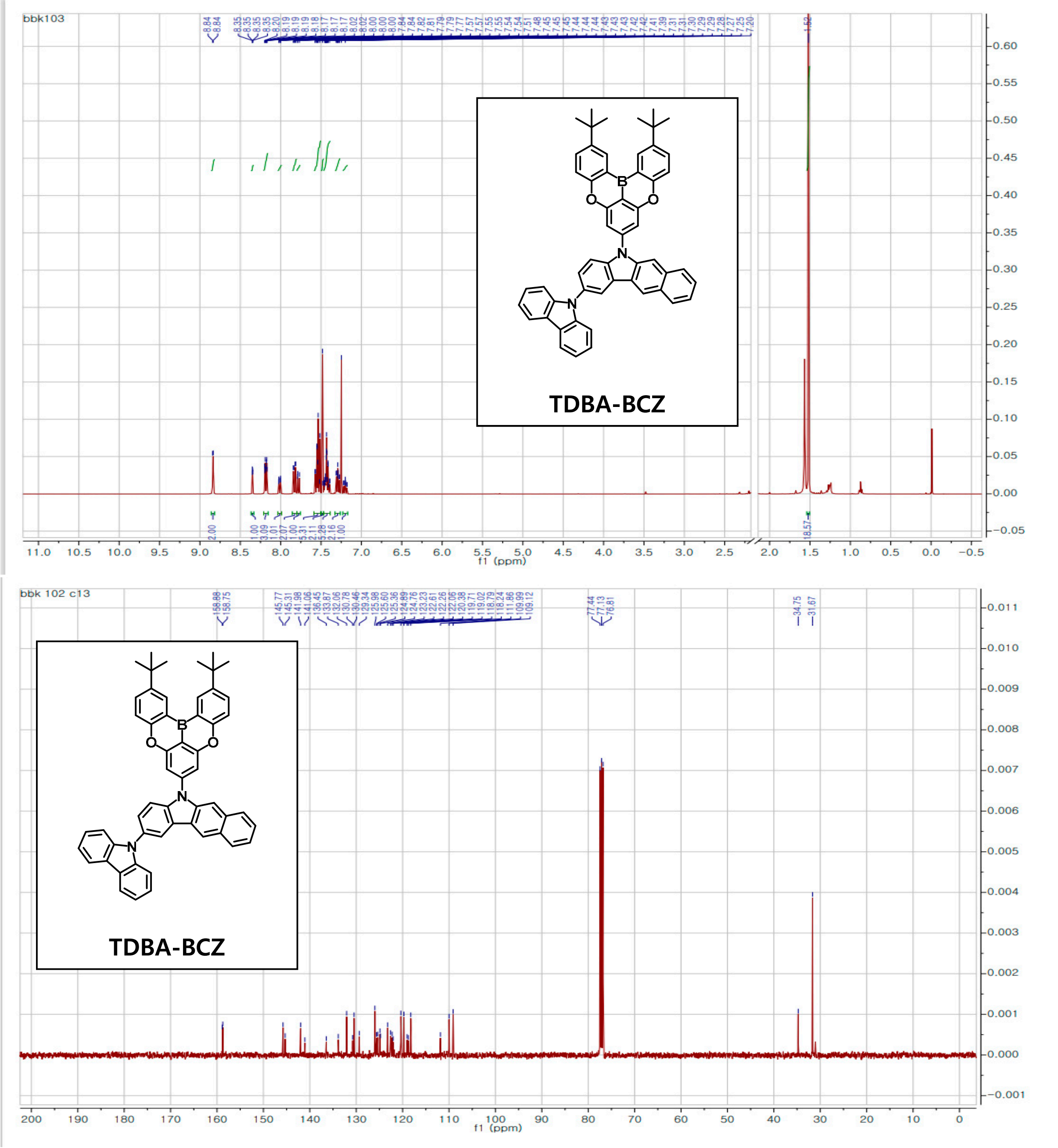

Figure. S11 ^{1}H-NMR (up) and ^{13}C-NMR (down) spectra of TDBA-BCZ.

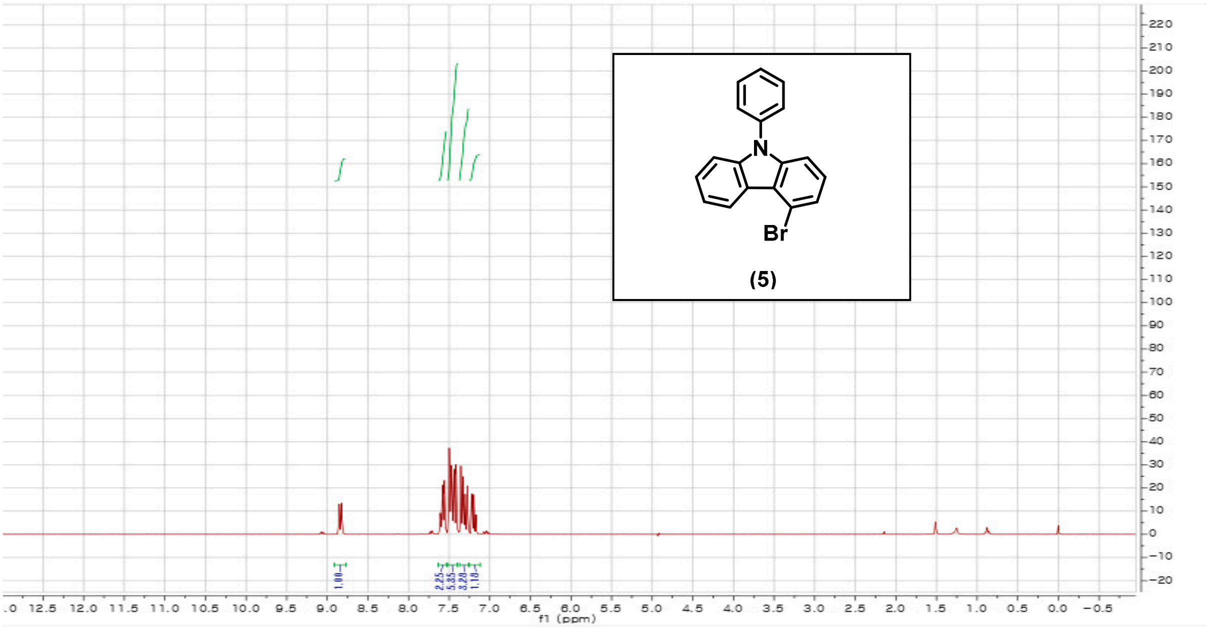

Figure. S12 ^{1}H-NMR spectrum of compound **5**.

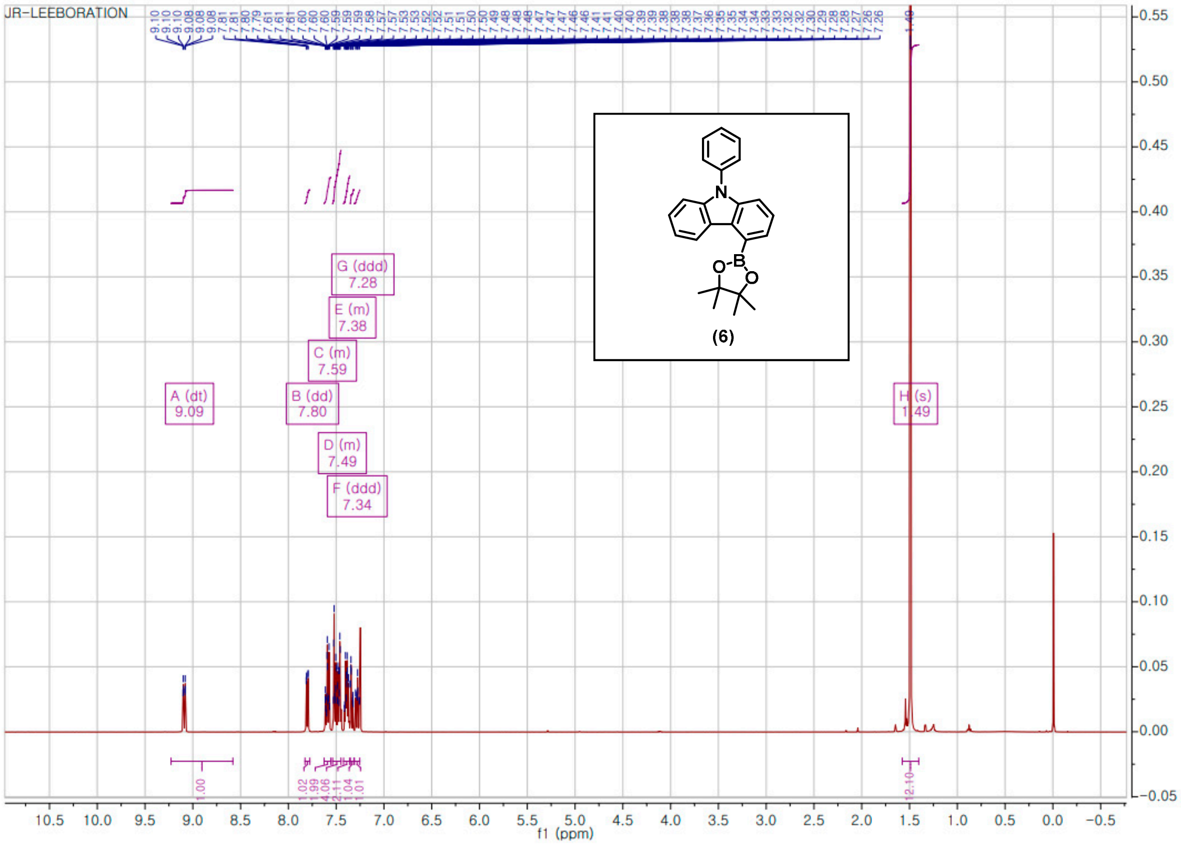

Figure. S13 ^{1}H-NMR spectrum of compound **6**.

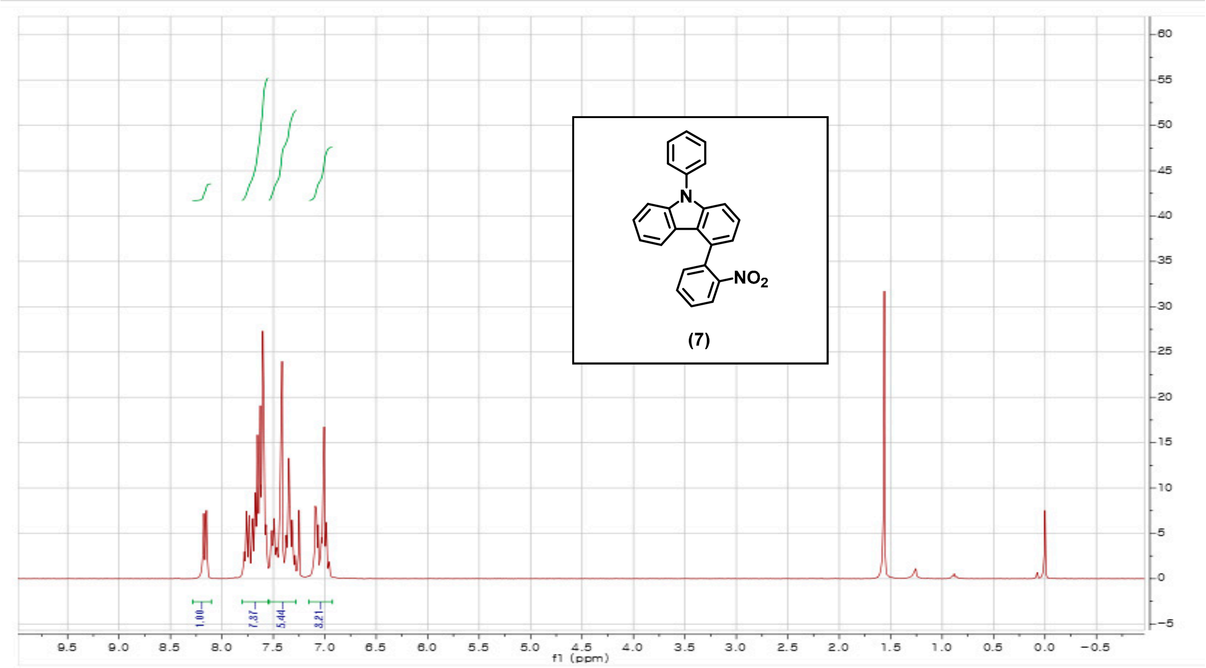

Figure. S14 ^{1}H-NMR spectrum of compound **7**.

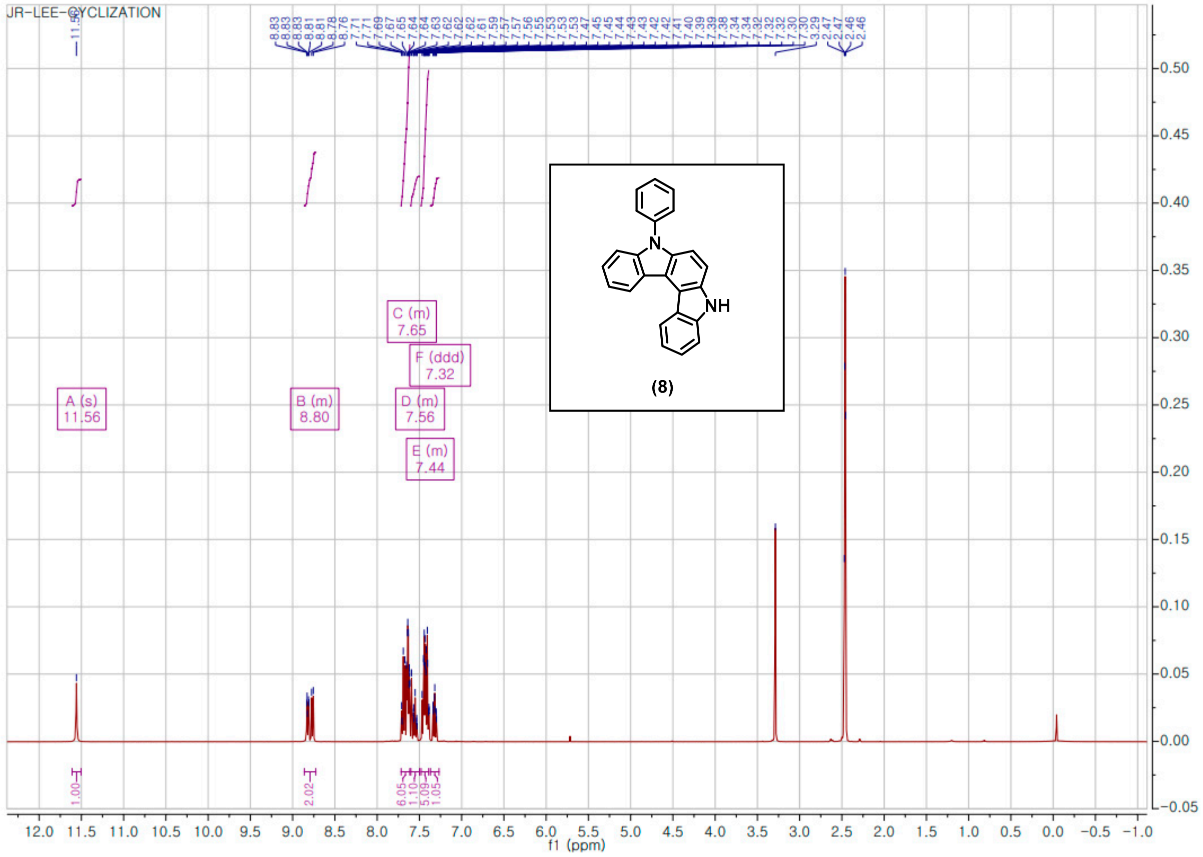

Figure. S15 ^{1}H-NMR spectrum of compound **8**.

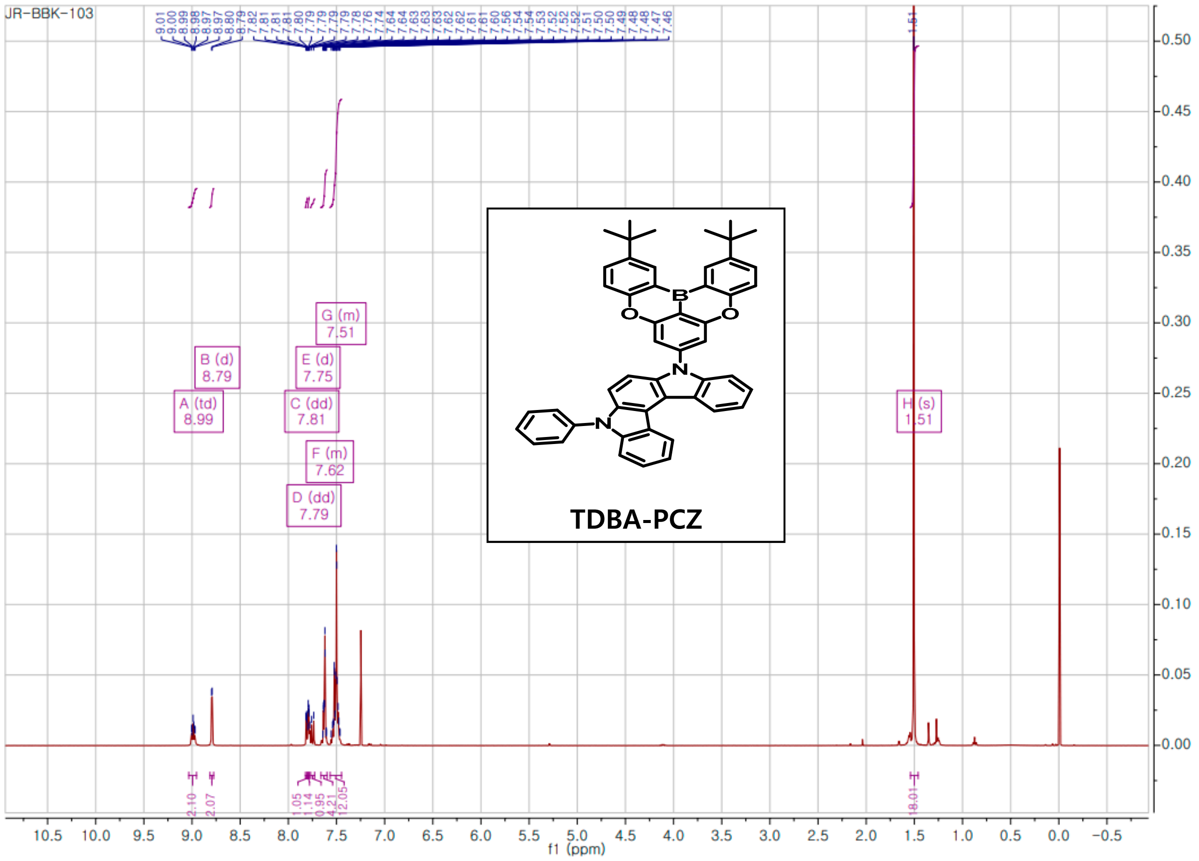

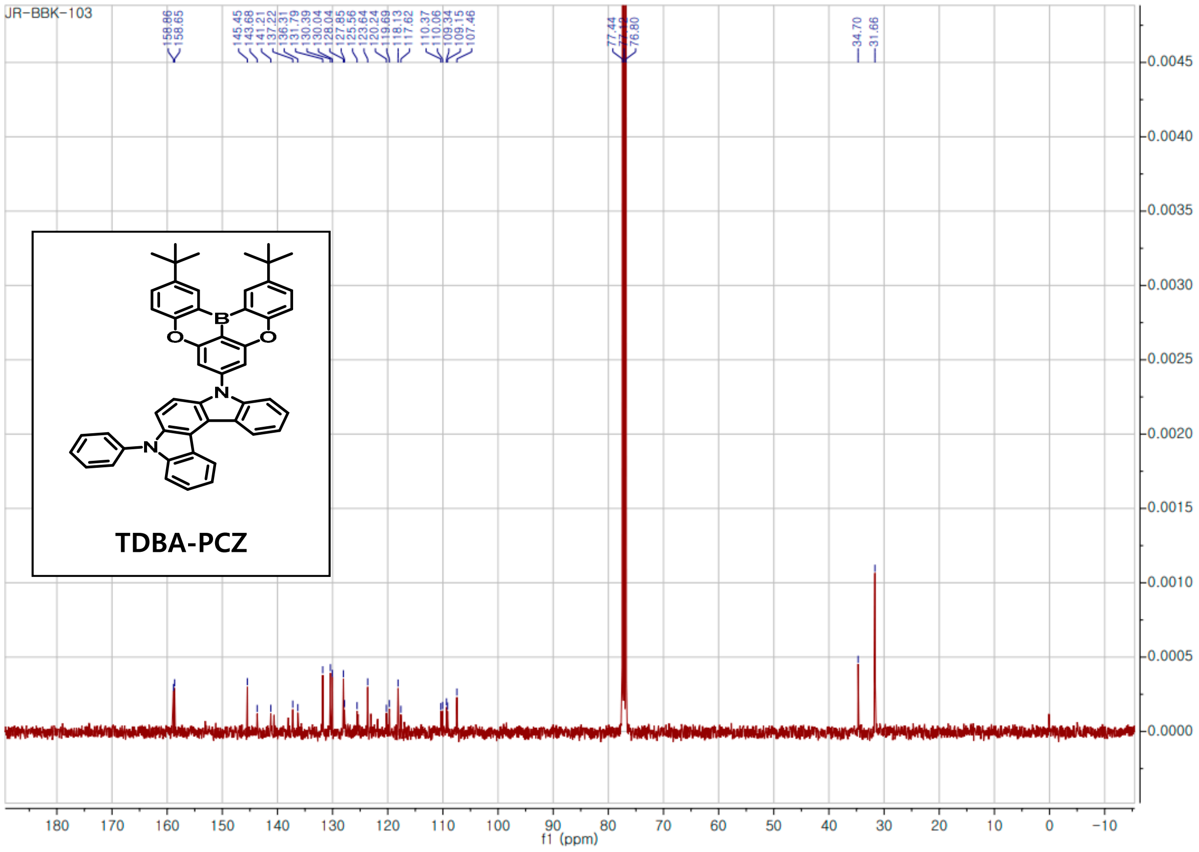

Figure. S16 ^{1}H-NMR (top) and ^{13}C-NMR (bottom) spectra of TDBA-PCZ.

Cross-sectional SEM image

We measured the cross-sectional SEM images of the multilayers in the conventional forward (a) and the inverted (b) OLED devices as shown in Figure. S17. Multilayers of (a) and (b) are PEDOT:PSS (40 nm)/PVK (20 nm)/mCP: 30% dopant (TDBA-PCZ) (20 nm)/TPBi (40 nm)/LiF (1 nm) and ZnO (30 nm)/PEI (5 nm)/mCP:30 wt% TDBA-PCZ (20 nm)/TAPC (20 nm)/MoO_3 (10 nm) for Figure. S17 (a) and (b), respectively. Looking at Figure. S17 (a), PEDOT:PSS with hydrophilic property shows the separated layer interface with 40.3 nm thickness and all other layers do not show the clear interfaces in picture because of the similar hydrophobic property. In the case of Figure. S17 (b), it does not show clear interface results between all layers, either. It can be explained by the fact that all layers do not have quite different hydrophobic properties.

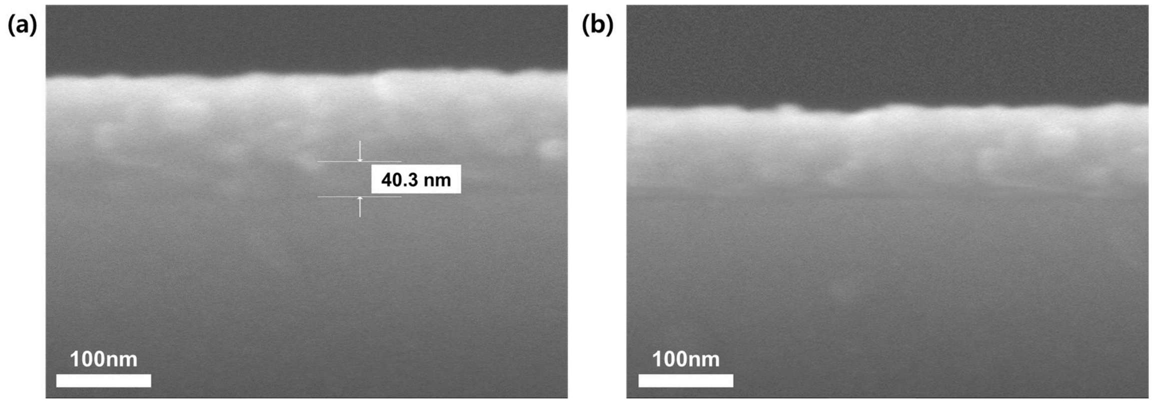

Figure. S17 Cross-sectional SEM image of (a) conventional forward device and (b) inverted device.
